# Cognitive and psychological dysfunction is present after a first seizure, prior to epilepsy diagnosis and treatment at a First Seizure Clinic

**DOI:** 10.1002/epi4.12909

**Published:** 2024-02-06

**Authors:** Remy Pugh, David N. Vaughan, Graeme D. Jackson, Jennie Ponsford, Chris Tailby

**Affiliations:** ^1^ School of Psychological Sciences Monash University Melbourne Victoria Australia; ^2^ Florey Institute of Neuroscience and Mental Health Melbourne Victoria Australia; ^3^ Department of Neurology Austin Health Melbourne Victoria Australia; ^4^ Monash Epworth Rehabilitation Research Centre Epworth Healthcare Melbourne Victoria Australia; ^5^ Department of Clinical Neuropsychology Austin Health Melbourne Victoria Australia

**Keywords:** cognition, depression, epilepsy, memory, telehealth

## Abstract

**Objective:**

Neuropsychological comorbidities found in chronic epilepsy have also been reported earlier in the disease course. However, recurrent seizures, antiseizure medication (ASM), and adjustment to a chronic diagnosis remain potential confounds of this literature. It thus remains unclear whether these comorbidities are primary or secondary attributes of epilepsy. To capture individuals as close to disease onset as possible, we studied the cognitive and psychological functioning in adults after their first seizure, yet prior to epilepsy diagnosis and treatment.

**Methods:**

Using a telehealth‐based prospective design, we screened cognition, mood, and anxiety symptoms in adult patients referred to a First Seizure Clinic (FSC), who were over 18 years, English‐speaking and not taking ASM. We screened cognition via telephone, and psychological symptoms via online questionnaires, all prior to the patients' diagnostic evaluation. Data were collected on 32 individuals subsequently diagnosed with epilepsy at the FSC, and 30 healthy controls from the community, who were matched to the epilepsy group for age, gender, and education.

**Results:**

A multivariate analysis of variance revealed that the groups differed significantly on combined cognitive measures with a large effect size (*F*_[1,56]_ = 5.75, *p* < 0.001, *η* ^2^ = 0.45). Post‐hoc analyses showed that performances on measures of verbal memory, working memory, and executive functions were significantly worse for the newly diagnosed epilepsy group than controls. The epilepsy group also exhibited higher rates of clinically significant depressive and anxiety symptoms.

**Significance:**

Cognitive and psychological dysfunction is prevalent in people with epilepsy as early as the first seizure event, before the influence of diagnosis, ASM and recurrent seizures. Their neuropsychological profile parallels that seen in chronic epilepsy, showing that this dysfunction is already present at the very onset of the disease. The current study demonstrates the viability of telehealth neuropsychological screening for all new epilepsy cases.

**Plain Language Statement:**

The results of this study show, using telephone‐based cognitive assessment and online questionnaires, that people with newly diagnosed epilepsy can experience problems with their thinking and memory skills, and low mood and anxiety, as early as after their first seizure. These issues are apparent at the very beginning of the disease, before an epilepsy diagnosis is made and before antiseizure medication is commenced, which suggests that they are due to the underlying brain disturbance, rather than the secondary effects of seizures, treatment, or lifestyle changes. Telehealth‐screening of thinking skills and mental health for all new epilepsy cases is recommended to promote early management of such problems.

**Key points**

- Cognitive and psychological morbidity is high among people with new‐onset epilepsy.
- We demonstrate that cognitive and psychological problems are prevalent after first seizure, even prior to diagnosis and treatment initiation.
- These deficits are present at disease onset, in the absence of ASM use/polypharmacy and recurrent seizures that characterize chronic cohorts.

## 1 INTRODUCTION

Cognitive and psychological comorbidities are core features of the epilepsies.^1^ Specifically, impairments in memory, attention, language and executive functioning are well‐documented across epilepsy syndromes,^2^, ^3^, ^4^ as are high rates of mood and anxiety disorders.^5^ Much of what we know about these neuropsychological comorbidities comes from the study of chronic epilepsy.^6^ However, research in these cohorts is often complicated by the presence of multiple factors that can influence neuropsychological function, confounding interpretation as to what is intrinsic to the disease versus what is secondary to chronicity and treatments. In particular, antiseizure medications (ASM) can negatively impact cognition and psychological function,^7^, ^8^ with polytherapy (multiple ASM) predicting worse cognitive outcomes.^8^, ^9^, ^10^ Recurrent seizures, and especially generalized tonic–clonic seizures and status epilepticus, have also been linked to poorer cognitive outcomes.^2^, ^11^, ^12^, ^13^

It is virtually impossible to disentangle the unique contributions of these risk factors in chronic epilepsy. As such, there has been growing interest in studying new‐onset epilepsy in an attempt to remove the influence of confounds and help elucidate the natural course of neuropsychological deficits.^14^ Accordingly, emerging evidence has demonstrated cognitive impairments in a high proportion of adults with newly diagnosed epilepsy. This includes studies both with individuals who have already commenced ASM,^15^, ^16^, ^17^ and in those who are yet to commence such treatment, with up to 50% of medication‐naïve cohorts already cognitively compromised.^13^, ^18^, ^19^, ^20^ The most commonly affected domains among these studies include memory, processing speed, attention, and executive functions. While these findings imply the presence of neuropsychological deficits at or before disease onset, these samples were evaluated several months after diagnosis and up to years since the first seizure. This timeframe allows ample opportunity for recurrent seizures and interictal activity to occur, which may influence the results. For instance, of two large studies that captured neuropsychological status prior to ASM commencement, one indicated a median of 555 days and nine seizures prior to assessment,^18^ and another noted mean values of 92 days between epilepsy onset and testing, and a seizure frequency of 4.1 per 60 days.^13^ Further, the psychological adjustment to receiving a formal epilepsy diagnosis has shown to be significant,^13^, ^21^ and this emotional distress (e.g., low mood, anxiety) could also negatively impact cognitive performance.^22^

There is a need to explore the neuropsychology of epilepsy as close to seizure onset as possible to minimize these additional influences. Such an investigation is challenging as there is typically only a short window of opportunity to capture individuals between first seizure and subsequent diagnosis and treatment. Furthermore, comprehensive neuropsychological assessment for every new‐onset epilepsy patient is not a feasible reality in current clinical practice. Here we adopted a neuropsychological screening approach^23^ to address this challenge. Tele‐neuropsychology offers an efficient, low‐burden, and low‐cost opportunity to screen for neuropsychological difficulties. It also improves reach to patients by reducing geographical barriers, eliminating travel costs for patients (many with likely driving suspensions following their seizure event), and minimizes infection exposure.^24^

The current study aimed to capture the neuropsychological profile of drug‐naïve new‐onset epilepsy after first seizure yet *before* diagnostic evaluation, achieving this via telephone and online questionnaire‐based neuropsychological screening. We hypothesized that patients with new‐onset epilepsy would show cognitive and psychological dysfunction prior to diagnosis and treatment, relative to neurologically healthy controls.

## 2 MATERIALS AND METHODS

### 2.1 Design

Patients were prospectively recruited from referrals to the Austin Health First Seizure Clinic (FSC), a diagnostic neurology clinic in Melbourne, Australia, involved in assessment and treatment of those presenting with a first suspected seizure. Following referral to the FSC, participants were recruited and screened promptly after their first seizure event, prior to their initial FSC evaluation at which diagnosis and management occurs. The study was approved by the Austin Health Human Research Ethics Committee (HREC: 59148).

### 2.2 Participants

#### 2.2.1 Patients

Potential participants were contacted via phone and introduced to the study. Inclusion criteria included aged 18 years and older, English‐speaking and not taking ASM. Individuals who were deemed unlikely able to complete telephone‐based assessment independently, for example, due to hearing impairment or moderate–severe intellectual/neurocognitive disability, were excluded. Those providing informed consent were invited to complete psychological screening questionnaires online and cognitive screening measures via phone, all prior to their initial FSC visit. Telephone cognitive screening was conducted by trained neuropsychologists in a standardized manner. Patient electronic medical records were monitored thereafter to collect prospective data on diagnostic status. The clinical diagnosis of epilepsy was made by a qualified neurologist within the FSC according to the International League Against Epilepsy (ILAE) criteria, with focus on a thorough clinical history, eyewitness accounts, outpatient EEG, and epilepsy‐directed MRI‐brain investigations. We collected data on 32 individuals who received a subsequent diagnosis of epilepsy (either at initial or subsequent FSC visit), recruited between February 2021 and July 2023.

#### 2.2.2 Controls

Control participants were identified as healthy volunteers who responded to study flyers advertised in local community centers and on social media. Exclusion criteria for controls included past or current neurological condition, current psychiatric condition, and current use of psychotropics or ASM. Of 65 potential controls, five were excluded due to having psychological or neurological conditions, four were uncontactable following initial contact, and three withdrew from the study due to time constraints. In the remaining 53 controls who underwent screening, there was an overrepresentation of females and those with tertiary education degrees. We therefore selected a subset who were matched to the epilepsy group on age, gender, and education, yielding a total of 30 controls for the analysis (see Table 1). The selection of these 30 cases was based solely on demographic factors, without reference to cognitive or psychological scores.

**TABLE 1:** Sample characteristics for epilepsy and control groups.

	Epilepsy	Controls	*p*
*N*	32	30	
Male/female, *N*	19/13	16/14	0.63
Age at testing	44.66	45.03	0.94
Level of education			
<=12 years	17	14	0.60
Tertiary degree	9	13	
Postgraduate degree	3	3	
Epilepsy type			
Focal	20		
Generalized	5		
Unknown	7		
Tonic–clonic seizure, *N*	26		
History of undiagnosed seizures prior to the index event, *N*	11		
Epileptogenic lesion on MRI, *N*	7		
Days between index seizure and cognitive screening, *M* (range)	21.21 (2–79)		

### 2.3 Measures

Cognitive and psychological measures were selected to reflect domains that are commonly affected in epilepsy, and sensitive to impairments in epilepsy. To increase accessibility and maximize the likelihood of recruitment within the short window between FSC referral and clinical appointment, we developed a tailored cognitive screening battery administrable via telephone. Such a telephone‐based approach has been used in other clinical populations.^25^, ^26^

We assessed verbal learning and memory using a modified version of the Rey Auditory Verbal Learning Test (RAVLT),^27^ consisting of three learning trials plus a free recall delay trial administered 20–30 min after learning. Attention, working memory and executive functions were assessed using: the Wechsler Adult Intelligence Scale 4th Edition (WAIS‐IV) Digit Span and Arithmetic subtests (deriving the Working Memory Index, WMI), Serial 3 s (i.e., counting backwards from 100 by 3 s), the Oral Trail Making Test (OTMT), Controlled Oral Word Association Test (COWAT), and the Delis‐Kaplan Executive Function System (D‐KEFS) Category Switching (accuracy score). Total administration time for the telephone battery was approximately 30 min.

Psychological functioning was assessed using the Neurological Disorders Depression Inventory for Epilepsy (NDDI‐E)^28^ for self‐reported depressive symptoms, and the Brief Epilepsy Anxiety Survey Instrument (brEASI)^29^ for anxiety symptoms. These questionnaires were administered via online surveys, using REDCap.^30^ Cognitive and psychological data were collected prior to the FSC visit.

### 2.4 Statistical analyses

Independent samples *t*‐tests and chi‐squared tests were used to examine demographic differences between the epilepsy and control groups. Regarding cognitive functioning, a between‐subjects multivariate analysis of variance (MANOVA) was conducted to determine whether groups differed on the combined cognitive variables: RAVLT Learning Total (total words recalled across trials 1–3), total words recalled at RAVLT 20‐min Delay, WMI (age‐corrected scaled score), Serial 3 s (total number of correct subtractions), OTMT‐B completion time, total number of valid words on the COWAT, and Category Switching Accuracy (age‐corrected scaled score). To ensure completeness of data, four epilepsy cases with missing values on the D‐KEFS Category Switching and two of which with missing Serial 3 s scores (due to introducing these tasks later in the protocol) were imputed with the epilepsy group means. One missing value on the Serial 3 s task for a control case by reason of technical disruption was also imputed with the control group mean. Three epilepsy cases who did not undergo cognitive screening and one epilepsy case with multiple unreliable scores due to participant misunderstanding were omitted from the analysis leaving *N* = 58 cases with complete data. Two epilepsy cases with univariate outliers on the OTMT‐B due to significant difficulty completing the task were winsorized. There were no multivariate outliers detected. One variable (OTMT‐B Time) with positive skew and kurtosis was log transformed for both patient and control groups. Results of evaluation of assumptions of homogeneity of variance–covariance matrices, linearity, and multicollinearity (i.e., none exceeding 0.9) were satisfactory. Univariate post‐hoc t‐tests were subsequently performed to explore unique effects of each cognitive variable by group. Cohen's *d* effect sizes and Bayesian statistics were also calculated. While the primary analysis was the MANOVA omnibus test, Benjamini–Hochberg corrections were applied to the post‐hoc t‐tests to adjust for multiple comparisons.

For psychological variables, rates of impairment between groups were analyzed with contingency tables using established clinical cut‐scores; greater than 15 on the NDDI‐E indicating high risk for Major Depressive Disorder (MDD)^28^ and at least 7 on the brEASI indicating high risk for any anxiety disorder (e.g., Generalized Anxiety Disorder, Social Anxiety Disorder, Specific Phobia, etc.).^29^ As the measures have been developed for detecting psychopathology in epilepsy cohorts, one question on the brEASI (“I avoided places where it would be difficult to get help if I had a seizure”) was omitted from the control questionnaire. Accordingly, predicted 8‐item scores were derived from the controls' 7‐item brEASI responses using a bivariate regression model, to compare groups. Rates of clinically significant psychological symptoms were evaluated for significance (*p* < 0.05) using chi‐square tests. All analyses were conducted using R/RStudio.

## 3 RESULTS

### 3.1 Sample characteristics

As given in Table 1, there were no statistically significant differences between participant groups on age, gender, and level of education (*p* < 0.05). In the epilepsy group, 20 were diagnosed with focal epilepsy, five with generalized epilepsy and in seven the type was unknown. The majority (21 of 32) had no history of seizures prior to the index event that prompted referral to the FSC. In 26 of 32 cases, the index event was a tonic–clonic seizure. All participants were assessed more than 48 h after their most recent seizure, with only three participants screened within 5 days of the index seizure. An epileptogenic lesion was identified on MRI in only six of 32 cases.

### 3.2 Cognitive functioning

#### 3.2.1 Degree of impairment between patients and controls

A between‐subjects MANOVA was conducted to explore whether epilepsy and control groups differed on the combined cognitive outcome variables. Using Pillai's trace criterion, the MANOVA showed that the groups differed significantly (*F*_[1,56]_ = 5.72, *p* < 0.001) with a large effect size of group on the combined DVs overall (η_p_^2^ = 0.44, 95% CI [0.22, 1.00]). The same effect was observed when running the MANOVA without cases with imputed data (*F*_[1,51]_ = 4.73, *p* < 0.001). Post‐hoc t‐tests revealed that scores on RAVLT Learning Total (*p* < 0.001), RAVLT 20‐minute delay (*p* < 0.001), WMI (*p* = 0.002), COWAT (*p* < 0.001), Category Switching (*p* = 0.006), and Serial 3 s (*p* = 0.005) were significantly worse in the epilepsy group than controls (see Table 2). These results remained robust after applying the Benjamini–Hochberg correction for multiple comparisons.

**TABLE 2:** Summary statistics for cognitive and psychological tests by group.

Cognitive Functioning						
MANOVA	*df*	*Pillai*	*f*	*p* Value	Partial *η* ^2^	
Group (Epilepsy/control)	1, 56	0.44	5.72	<0.001***	0.44	
Post‐hoc *t*‐tests	Epilepsy (*n* = 28), *M* (SD)	Control (*n* = 30), *M* (SD)		*p* Value	*d*	Bayes Factor
RAVLT learning total	20.75 (6.03)	28.47 (4.34)		<0.001***	1.48	20 649.95
RAVLT 20‐min delay	6.07 (3.45)	8.90 (2.62)		<0.001***	0.93	35.97
WMI	94.54 (13.56)	106.63 (14.92)		0.002**	0.85	16.65
COWAT	33.39 (13.82)	48.03 (12.94)		<0.0001***	1.09	206.87
Category switching accuracy SS	9.12 (3.77)	12.10 (4.24)		0.006**	0.74	6.60
OTMT‐B time	48.23 (30.18)	37.02 (19.33)		0.22	0.33	0.50
Serial 3s (*N* correct subtractions)	13.12 (8.02)	19.14 (7.48)		0.005**	0.78	8.93
**Psychological Functioning**						
	Epilepsy (*n* = 29)	Control (*n* = 31)		*p* Value	*d*	Bayes Factor
NDDI‐E, Median (IQR)	13.00 (8.00)	11.00 (3.75)		0.10	0.47	1.00
High‐risk score (>15), *N* (%)	9 (31)	2 (7)		0.02*		
brEASI, Median (IQR)	5.00 (8.00)	2.38 (2.19)^a^		0.14	0.64	3.07
High‐risk score (> = 7), *N* (%)	10 (34)	3 (10)		0.03*		

*Note*: ***significant at the <0.001 level, **significant at the <0.01 level, *significant at the <0.05 level.

Abbreviations: ANOVA, analysis of variance; brEASI, Brief Epilepsy Anxiety Survey Instrument; COWAT, Controlled Oral Word Association Test; D‐KEFS, Delis Kaplan Executive Functioning System; M, mean; MANOVA, multivariate analysis of variance; NDDI‐E, Neurological Disorders Depression Inventory for Epilepsy; OTMT‐B, Oral Trail Making Test – Part B; RAVLT, Rey Auditory Verbal Learning Test; SD, standard deviation; SS, Scaled Score; WMI, Working Memory Index.

^a^ Predicted 7‐item score.

In a secondary analysis, we repeated the MANOVA, including the NDDI‐E and brEASI as covariates, given the potential impact of mood and anxiety on cognitive scores.^31^ The between‐group cognitive effects remained significant under this model (*F*_[1,51]_ = 5.18, *p* < 0.001), implying that the cognitive differences were present over and above any potential influence of mood and anxiety.

#### 3.2.2 Rates of impairment

We also examined rates of cognitive impairment, defined as a z‐score less than −1.28 (i.e., 10th percentile) relative to the control group. In the epilepsy group, 86% (95% CI [0.67,0.95]) were impaired on at least one cognitive measure compared with 47% (95% CI [0.29,0.65]) of controls. Regarding verbal memory, 48% (95% CI [0.30,0.67]) of the epilepsy group demonstrated impaired scores across both the RAVLT Learning and Delay trials, relative to 3% (95% CI [0.00,0.19]) of controls. Regarding attention, working memory, and executive functions, 41% (95% CI [0.24,0.61]) of the epilepsy group were impaired on at least two measures, compared with 10% (95% CI [0.03,0.28]) of controls.

### 3.3 Psychological functioning

On the NDDI‐E, 31% of epilepsy participants were at high risk for MDD compared with 6% for controls (*p* = 0.02). On the brEASI, 34.5% of the epilepsy group were at high risk for any anxiety disorder compared with 10% for controls (*p* = 0.03). Regarding the severity of symptoms, non‐parametric Mann–Whitney U tests showed that median scores on both the NDDI‐E and brEASI were not statistically significantly different between groups (*p* > 0.05), as shown in Table 2.

## 4 DISCUSSION

The present study aimed to prospectively characterize cognitive and psychological function in people with new‐onset epilepsy after first seizure, prior to diagnosis and treatment. Our methodological approach – using telephone‐based cognitive testing and online questionnaires – allowed for investigation of neuropsychological functioning as soon after seizure onset as practically possible, minimizing the effect of typical confounds present in previous studies, such as recurrent seizures and the psychological impact of an epilepsy diagnosis.

Our hypothesis that people with epilepsy would show cognitive and psychological dysfunction relative to their neurologically healthy peers was supported. In particular, we found that adults with new‐onset epilepsy were cognitively compromised in the domains of verbal learning and memory, working memory and executive functioning after first seizure, before diagnosis and treatment initiation. The largest effects were seen for verbal learning and memory. The results remained significant after accounting for mood state.

### 4.1 Cognitive functioning

The pattern of cognitive impairment found is similar to that observed in chronic epilepsy.^2^, ^32^, ^33^ Deficits in memory, attention, and executive functions have also been found in other new‐onset/new‐diagnosis epilepsy studies.^13^, ^14^, ^16^, ^19^, ^34^ Our results are most comparable with other new‐onset, medication‐naïve cohorts assessed following diagnosis, which show such deficits in up to 50% of cases.^13^, ^18^, ^20^ Precise comparison of the degree of cognitive impairment between new‐onset studies is, however, difficult given the significant methodological variability across samples. Specifically, differences relate to sample characteristics (e.g., inclusion or exclusion of those with neurological and/or psychological comorbidities, sample size), variation in cut‐scores used to define impairment (e.g., −1 or − 2 SD), and differences in outcome measures. Further, some studies do not report the underlying distributions of the data. We also note that several new‐onset studies relied upon normative data alone, rather than control groups of similar age, gender and education.^16^, ^20^, ^35^, ^36^ However, despite these differences, there seems to be a convergence of findings that neuropsychological deficits are present at disease onset, with the nature of deficits resembling that of chronic epilepsy.

### 4.2 Psychological functioning

We found that a greater proportion of people with new‐onset epilepsy had clinically significant anxiety and depressive symptomatology than did controls, with approximately one third of patients at high risk each for MDD and a DSM‐V anxiety disorder. The current findings in new‐onset cases are consistent with those seen in chronic epilepsy^37^ as well as other newly diagnosed cohorts.^16^, ^38^, ^39^ Regarding overall symptom burden, there was a trend for people with epilepsy to report higher levels of depression and anxiety; however, this was not significantly different to controls. Overall, our results are clinically important, as those with high depressive symptoms around the time of diagnosis have been shown to remain distressed in the following year.^40^ Furthermore, there is evidence to suggest that psychological dysfunction increases the risk of seizure recurrence.^21^

### 4.3 Potential basis of cognitive and psychological dysfunction at disease onset

What might explain the cognitive and psychological dysfunction in this newly diagnosed, drug‐naïve cohort? The majority of our cases were MRI‐negative (25 of 32 cases) and had only experienced a single seizure prior to testing (21 of 32 cases). Therefore, the results cannot be attributed to macroscopic structural brain abnormalities nor cumulative seizure effects. In addition, cognitive screening was conducted at least 48 h after the seizure event. The literature suggests that post‐ictal effects usually resolve within 24 h of the seizure,^41^ and therefore we do not believe the findings are influenced by this factor. In this regard, we suggest two potential drivers of the findings. Firstly, these deficits present from disease onset likely reflect, and are symptomatic of, the underlying network disturbance that is also responsible for generating seizures.^42^, ^43^ That is, while seizures represent a dramatic paroxysmal manifestation of altered brain activity within neural networks, we assume that a degree of network disruption remains present in the interictal period giving rise to cognitive and psychological dysfunction. Relatedly, cognitive deficits may also result from network reorganization hypothesized to represent a homeostatic adaptation geared toward containing cortical excitability, thereby minimizing the potential for seizures in the presence of aberrant epileptic activity.^44^ In light of previous findings,^8^, ^10^, ^11^ it is then possible that factors such as ASM and recurring seizures may exacerbate neuropsychological deficits. Alternatively, ASM may have a protective effect on cognition in some cases via suppression of epileptic activity.

It should be noted that we chose to screen people prior to clinical evaluation to lessen the impact of the epilepsy diagnosis on neuropsychological outcomes via psychological distress, which can be marked.^13^ However, we could not empirically account for the potential impact of the first seizure experience itself on such functioning, which represents an uncertain and distressing event for some individuals.^21^ In future work, we plan to compare neuropsychological functioning in our new‐onset epilepsy group with other pre‐diagnosis FSC groups (e.g., acute symptomatic seizures, seizure mimics) who are matched for this situational uncertainty. Studying this broader cohort may assist in determining whether the pattern of our results is unique to epilepsy or not, and the extent to which the results of screening provide prognostic information. Nevertheless, the parallels between our own findings and those in more chronic cohorts suggests that neuropsychological morbidity observed here does not simply reflect an acute reactional state.

### 4.4 Utility of telehealth‐based neuropsychological screening in a first seizure setting

The current findings highlight the need for neuropsychological screening at disease onset, preferably prior to treatment, firstly to promote early identification and intervention of deficits, and secondly, to establish a baseline with which to compare post‐treatment functioning. Objective screening is particularly important as individuals with newly diagnosed epilepsy tend to underestimate cognitive deficits.^13^, ^20^ As experienced in our study, feedback of neuropsychological results to the clinic prior to patients' initial evaluation has the potential to guide formulation and management, and bring neuropsychological issues to the foreground early. Neuropsychological screening may facilitate appropriate ASM choice based on the individual's cognitive and psychological profile. For example, it may be desirable to avoid ASM posing high cognitive risk (e.g., topiramate) for individuals with already compromised cognition, to select ASM with mood stabilizing properties and avoid ASMs with high‐risk of psychological side effects for those with high mental health symptomatology, or more generally, to use longitudinal monitoring to inform decisions about the balance of cognitive risk versus seizure control. Screening will also flag individuals in need of comprehensive neuropsychological evaluation.

### 4.5 Limitations and future directions

We have developed and demonstrated feasibility of a brief, cost‐effective tele‐neuropsychological screening battery that is widely accessible to patients of all ages and geographical locations. One challenge, however, was including a wide range of reliable telephone‐based measures of cognition that are sensitive in epilepsy. In contrast to our other findings, the OTMT‐B, a measure of divided attention and speed, did not significantly differentiate the groups. We suspect this is due to the nature of the oral version, given that its written form has shown sensitivity in epilepsy populations,^45^, ^46^, ^47^ including in new‐onset cohorts.^15^ Moreover, the broader literature has shown only modest correlations between the oral and written versions of the task.^48^ We were also unable to include a reliable measure of processing speed via telephone; a domain that is commonly affected in epilepsy.^2^, ^18^, ^49^ Development of such a task would be immensely useful for clinical and research neuropsychology. Our use of telephone administration also meant that participants could not be seen during screening, which may have increased the risk of cheating (e.g., writing down responses), although the incidence of such behavior has found to be low in other studies.^26^, ^50^ Our participants were explicitly instructed not to use external materials (pen/paper/devices) to assist their performance during the assessment. Lastly, we did not have a sufficient sample size to perform subgroup analyses on the different epilepsy etiologies or syndromes, which we would encourage in future studies. Nonetheless, it is clear in our sample that common neuropsychological deficits are present across a natural, heterogenous new‐onset population, as seen in chronic epilepsy.

## 5 CONCLUSION

In conclusion, our study indicates that cognitive and mood disturbances are prevalent at epilepsy onset, prior to diagnosis and treatment. Specifically, our telehealth‐screening approach allowed us to capture neuropsychological function as close to disease onset as practically possible, minimizing the confounds of recurrent seizures, ASM, and psychological adjustment to a chronic diagnosis. The cognitive disturbance was still present after accounting for mood and anxiety. Our findings contribute to the growing body of work suggesting that neuropsychological comorbidity is a primary attribute of epilepsy at onset. These findings highlight the viability of remote neuropsychological screening for all new presentations of epilepsy, preferably before medication commencement, to promote early identification and intervention.

## AUTHOR CONTRIBUTIONS

**Remy Pugh:** conceptualization; project investigation and administration (lead); formal analysis (lead); writing – original draft (lead). **David Vaughan:** resources (equal); writing – review and editing (equal). **Graeme Jackson:** resources (equal); writing – review and editing (equal). **Jennie Ponsford:** supervision (supporting); writing – review and editing (equal). **Chris Tailby:** conceptualization (lead); project administration; supervision (lead); writing – review and editing (lead).

## CONFLICT OF INTEREST STATEMENT

None of the authors has any conflict of interest to disclose. We confirm that we have read the Journal's position on issues involved in ethical publication and affirm that this report is consistent with those guidelines.
